# Enteric Viruses in Turkeys: A Systematic Review and Comparative Data Analysis

**DOI:** 10.3390/v17081037

**Published:** 2025-07-24

**Authors:** Anthony Loor-Giler, Sabrina Galdo-Novo, Luis Nuñez

**Affiliations:** 1 Laboratorios de Investigación, Dirección General de Investigación, Universidad de las Américas (UDLA), Antigua Vía a Nayón S/N, Quito EC 170124, Ecuador; a.abel.loor.giler@gmail.com; 2 Facultad de Ciencias Veterinarias, Universidad de Buenos Aires, Av. Chorroarín 280, Buenos Aires 1427, Argentina; sgaldonovo@fvet.uba.ar; 3 Facultad de Ciencias de la Salud, Carrera de Medicina Veterinaria, Universidad de Las Américas, Antigua Vía a Nayón S/N, Quito EC 170124, Ecuador; 4 One Health Research Group, Facultad de Ciencias de la Salud, Universidad de Las Américas, Antigua Vía a Nayón S/N, Quito EC 170124, Ecuador

**Keywords:** enteric, viruses, turkeys, PEC, prevalence

## Abstract

Enteric diseases represent one of the main causes of morbidity and mortality in poultry production, especially in turkeys (*Meleagris gallopavo*), significantly affecting the profitability of the sector. Turkey enteric complex (PEC) is a multifactorial syndrome characterized by diarrhea, stunting, poor feed conversion, and increased mortality in young turkeys. Its aetiologia includes multiple avian enteric viruses, including astrovirus, rotavirus, reovirus, parvovirus, adenovirus, and coronavirus, which can act singly or in co-infection, increasing clinical severity. This study performs a systematic review of the literature on these viruses and a meta-analysis of their prevalence in different regions of the world. Phylogenetic analyses were used to assess the genetic diversity of the main viruses and their geographical distribution. The results show a wide regional and genetic variability, which underlines the need for continuous epidemiological surveillance. Health and production implications are discussed, proposing control strategies based on biosecurity, targeted vaccination, and optimized nutrition. These findings highlight the importance of integrated management to mitigate the impact of CSF in poultry.

## 1. Introduction

Enteric diseases represent one of the main causes of morbidity and mortality in poultry production, particularly in turkeys (*Meleagris gallopavo*), with a significant impact on industry profitability [1]. Poult Enteric Complex (PEC) is a multifactorial syndrome characterized by diarrhea, growth retardation, decreased feed conversion, and increased mortality, especially in turkey poults during the first weeks of life [2]. Although its etiology is complex and involves multiple infectious agents and environmental factors, avian enteric viruses have been consistently identified as key players in the pathogenesis of the syndrome [2]. Depending on whether the infection is monocausal or multicausal, several syndromes within PEC have been reported, such as turkey enteritis and mortality syndrome (PEMS) [3], turkey mortality syndrome (TMS) [4], turkey runting-stunting syndrome (RSS) [5], light turkey syndrome (LTS) [6] and, most frequently, poult enteritis syndrome (PES) [7], which affects young poults from 1 to 7 days of age. The manifestation of any of these symptoms generates significant economic losses in poultry farms due to low feed conversion, weight reduction, and mortality [8].

Several viruses have been associated with PEC, including avian rotavirus (AvRV), avian astrovirus (AAstV), avian reovirus (ARV), avian parvovirus (APV), and to a lesser extent, avian calicivirus (ACV), avian adenovirus (AAdV), avian coronavirus (AvCoV), and avian picornavirus (AvPiV) [9,10]. These agents can act individually or in co-infection, exacerbating the severity of the clinical picture [11]. Viral coinfections are of particular concern, as they can increase the total viral load and favor the persistence of infection in poultry populations.

The prevalence and distribution of these viruses vary widely among regions and production systems, suggesting the influence of epidemiological and environmental factors on their transmission [8]. However, there remains a gap in the understanding of the global epidemiology. Considerable prevalence rates have been reported in some regions, while in others, little or no information is available, making a comprehensive assessment of the impact of these agents on poultry farming difficult [12,13,14,15]. In addition, genetic variability within certain viruses, such as avian astroviruses, rotaviruses, and coronaviruses, could influence their pathogenicity and transmission capacity, underscoring the importance of phylogenetic analyses in epidemiological studies [16,17,18,19,20,21,22].

Given this context, the present study aims to perform a systematic review of the literature on enteric viruses and other agents associated with PEC in turkeys, and a meta-analysis analyzing their prevalence in different geographical regions and comparing the available data by means of a descriptive statistical analysis. Additionally, the genetic diversity of the main viruses will be evaluated through a phylogenetic analysis, in order to identify distribution patterns and possible emerging lineages. Finally, the implications of these findings for avian health and poultry production will be discussed, proposing recommendations for future research and control strategies.

## 2. Materials and Methods

### 2.1. Literature Search Strategy

For the collection of information on the prevalence of enteric viruses in turkeys associated with Poult Enteric Complex (PEC), a systematic search was conducted in PubMed, Scopus, and Web of Science. Key terms combined with Boolean operators were used, such as “enteric virus” OR “avian enteric virus” OR “poult enteric complex” AND “prevalence” OR “incidence” OR “epidemiology” AND “Meleagris gallopavo” OR “turkey”, excluding studies on experimental vaccination or immunology. Articles were selected following predefined inclusion and exclusion criteria. For prevalence analysis, we considered those that reported prevalence or absence of detection of enteric viruses in turkeys, published in the last 15 years in indexed journals. Studies lacking quantifiable data, not focused specifically on turkeys, or without primary data were excluded. Duplicate and non-relevant records were removed, while a limited number of older studies were retained when providing foundational virological information, including early virus descriptions, in vivo infections, or full genome analyses. Mendeley v2.132.0 was used for reference management and duplicate removal, while initial screening was performed in a blinded manner with Rayyan v1.6.0. Relevant data, such as the virus detected, the country of origin of the study, the reported prevalence with its confidence interval when available, and the detection methods used, were extracted.

### 2.2. Meta-Analysis

Prevalence data were organized in a comparative table by region and country, standardizing the values reported in percentages to facilitate comparison. Descriptive measures were calculated as means and ranges by virus type and geographic region. To assess significant differences between regions, a chi-square test (χ^2^) was applied, while temporal trends in virus detection were explored using regression analysis when sufficient longitudinal data were available; all statistical analyses were performed using RStudio v2024.12.1. The confidence level for these tests was 95%.

### 2.3. Phylogenetic Analysis

A phylogenetic analysis of the sequences of the main enteric viruses reported in turkeys, based on the lineages or genotypes found in previous literature for each, was performed to indicate their phylogenetic distribution. Sequences of key genes from every studied virus were collected from the GenBank database, taking all of them for those viruses in which there are fewer than 20 sequences, and, in the case where there were more than 20, randomly selecting sequences from each region was used. Sequence alignment was performed with ClustalW in Geneious Prime v2.0.10 (https://www.geneious.com) (accessed on 13 July 2025), and phylogenetic tree construction was performed using the Maximum Likelihood statistics method in MEGA 11 [23], selecting the most appropriate nucleotide substitution models for each alignment and phylogeny test bootstrap model with 1000 replicates. The generated trees were visualized and edited to highlight clades based on the phylogenetic groups of interest.

## 3. Main Enteric Viruses Associated with PEC

### 3.1. Avian Astrovirus

AAstV in poultry are highly contagious enteric pathogens that mainly affect young birds, causing diarrhea, growth retardation, and increased mortality, which impacts poultry production [9]. They are transmitted by the fecal-oral route and can persist in the environment, favoring their dissemination, in addition to vertical transmission [24]. Turkey Astrovirus (TAstV) and avian nephritis virus (ANV) are two members of the *Astroviridae* family and genus *Avastrovirus* that have been associated with enteric disease in turkeys since 1980 [25,26]. These small, non-enveloped viruses have an RNA genome of up to 7.9 kb [27]. They have three ORFs: ORF1a, which codes for nonstructural proteins; ORF1b, which codes for RNA-dependent RNA polymerase (RdRp); ORF2, which codes for capsid proteins [28]. Due to the high variability of ORF2, TAstV has been classified into two types: TAstV-1, first described in 1980 [25], and TAstV-2, described in 2000 [29]. ANV, initially linked to enteritis and renal impairment in chickens, has also been identified in turkeys, resulting in analogous symptoms that contribute to nutrient malabsorption [27]. Infection by these viruses occurs in the deepest part of the lamina propria, the basal portion, and coincidentally in the crypts. It is this damage to the intestinal villi and the coincidental hyperplasia of the crypts that causes decreased nutrient absorption, decreased digestive enzymes, and consequently osmotic diarrhea [30,31,32]. Enteric symptoms caused by TAstVs are concentrated in the digestive tract, without specific characteristics, even in some cases associated with immunosuppression processes since viral particles were identified in the bursa and thymus [33,34].

**Figure 1:**
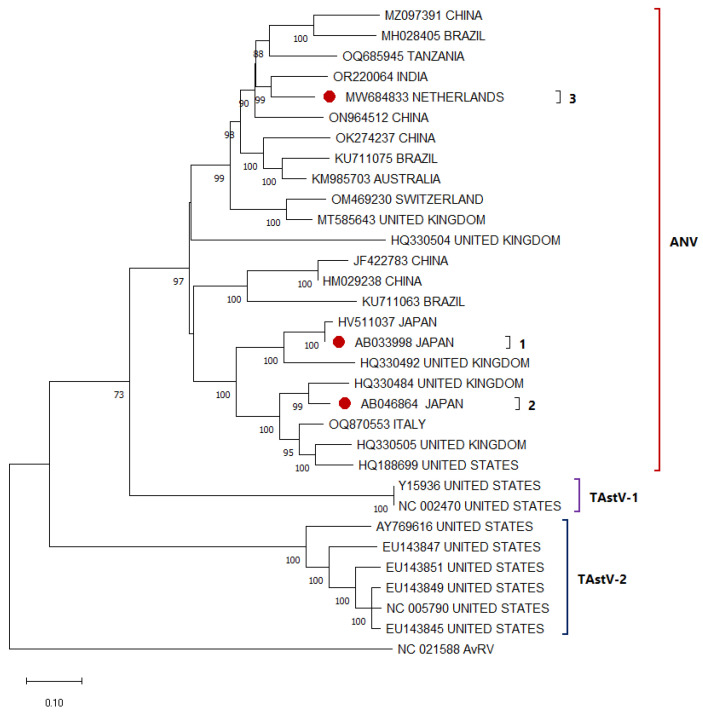
Phylogenetic relationships between AAstV nucleotide sequences (all reported sequences of TAstV-1 and 2 were included, and some sequences from every location were randomly selected) infecting turkeys (ANV in red bracket, TAstV-1 in purple bracket, TAstV-2 in blue bracket) based on ORF2 sequences previously deposited in the GenBANK distributed by the countries in which they were reported. The sequences were aligned using the CLUSTAL OMEGA method in Geneious Prime v2.0.10. (https://www.geneious.com) (accessed on 13 July 2025) software. The phylogenetic tree was constructed using the MEGA 11 software package using the maximum likelihood statistic method and the Tamura-Nei substitution model. The numbers along the branches refer to bootstrap values for 1000 replicates. The scale bar represents the number of substitutions per site. Reference sequences for ANV-1 to 3 were marked with a red circle. A sequence of AvRV (NC_021588) was used as an outgroup.

At least three serotypes of ANV [35] have been classified, with an additional serotype hypothesized. However, over the years, several genotypes (10 or more) have been reported, indicating that proper classification with serotypes has not been achieved. Even though clustering of sequences around the ANV 1–3 cluster is observed (Figure 1), it is not recognized that they generate the same pathogenic effect. While the two types of TAstVs (TAstV-1 and TAstV-2) have a high genetic divergence, categorizing them into two distinct clades (Figure 1), with divergence rates exceeding 50% in most instances. The specific pathogenic spectrum of each type has not been individually characterized, nor has the existence of subtypes within each been confirmed. Consequently, many studies have approached the identification of AAstV in a generalized manner. In the absence of detailed knowledge regarding the individual pathogenic effects, the presence of any of these viruses is considered a potential risk to poultry production [36]. Among the samples collected and pooled with TAstV-2 (AY769616), it is annotated as TAsTV-3 in GenBANK, as described in 2008 [36], because genotypic differences were found with strains previously described as TAstV-2. However, the nucleotide differences between this sequence and other TAstV-2 sequences are not sufficient to classify it as a new type, being only 20% and not close to 50% as with the other TAstVs. There are still not enough evolutionary or pathological analyses to separate it. Further and ongoing studies are needed to evaluate this hypothesis and determine the categorization of this apparent new TAstV.

### 3.2. Avian Rotavirus

Avian rotaviruses are viruses that affect a wide range of avian species and are mainly associated with diseases that affect growth, food absorption, and digestive health [37]. Transmission occurs primarily through contact with contaminated feces, where infectious material can persist in the environment for long periods of time [38]. Turkey rotavirus (TRotV) belongs to the family *Reoviridae* and the genus *Rotavirus*. It has a double-stranded RNA genome with 11 segments, coding for structural proteins (VP1-VP4, VP6, and VP7) and non-structural proteins (NSP1-NSP6) [39]. Based on the variations in the gene coding for VP6, the nine rotavirus groups (A-J) have been determined; however, only groups A, D, F, and G have been identified in birds [40]. Although these four types of rotaviruses have been identified in birds, Avian rotavirus A (AvRVA) is the most prevalent and associated with PEC [41]. Since their first identification in 1977 in turkeys with enteritis, AvRVA has presented a challenge to the poultry industry due to their symptomatology, characterized by watery diarrhea prior to other enteric symptoms [42]. In flocks of turkeys affected with enteritis, TRotV has been identified in up to 70% of individuals, usually accompanied by other enteric pathogens such as AAstV and ARVs [43,44].

**Figure 2:**
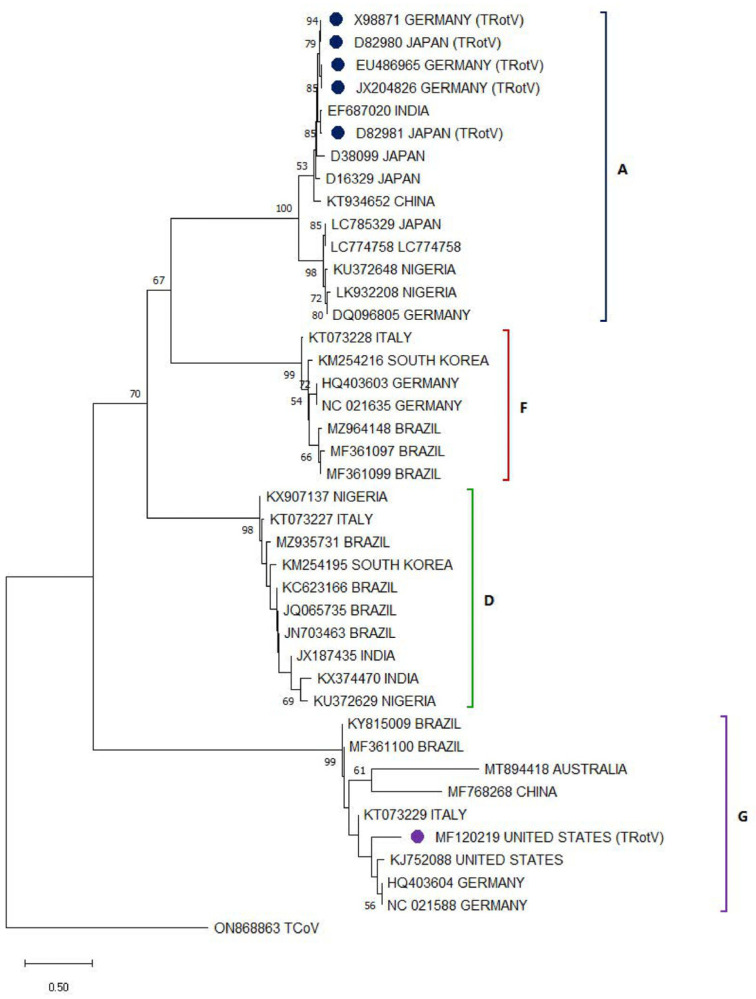
Phylogenetic relationships between AvRV nucleotide sequences (some sequences from every location were selected randomly), including sequences isolated in infected turkeys (type A in blue, F in red, D in green, and G in purple), based on VP6 sequences previously deposited in the GenBANK distributed by the countries in which they were reported. The sequences were aligned using the CLUSTAL OMEGA method in Geneious Prime v2.0.10 (https://www.geneious.com) (accessed on 13 July 2025). The phylogenetic tree was constructed using the MEGA 11 software package using the maximum likelihood statistic method and the Tamura-Nei substitution model. The numbers along the branches refer to bootstrap values for 1000 replicates. The scale bar represents the number of substitutions per site. TRotV sequences were marked with a circle. A sequence of TCoV (ON868863) was used as an outgroup.

Of the sequences deposited in the GenBANK of AvRV identified in turkeys, most correspond to group A [41], grouping together and moving away from AvRVA sequences identified in other birds, showing a genetic divergence between them (Figure 2). Although several studies have chosen to analyze the presence of all AvRV types, type A remains the most present and most associated with the occurrence of enteric disease. With the exception of a study in the United States that focuses on type G [45], its distribution and relationship with PEC, including the genetic difference of this sequence (MF120219) in comparison with AvRVG identified in other birds (Figure 2). Although rotaviruses infecting birds show genetic differences from those infecting humans, recombinant events between AvRV and Human rotavirus strains have recently been hypothesized [38,45]; therefore, recombinant strains with zoonotic potential from birds to humans have been hypothesized to appear in the future.

### 3.3. Avian Reovirus

Avian reoviruses are viruses that affect various avian species and are associated with enteric diseases that can compromise growth and joint health [46]. They are transmitted mainly by contact with fecal excretions, being able to survive for several weeks in water and turkey litter, and through eggs (vertical transmission), facilitating their spread in poultry [47]. Turkey reovirus (TRV) is a member of the family *Reoviridae* and the genus *Orthoreovirus*. It has a double-stranded RNA genome composed of 10 segments of different sizes: 3 L-segments (Large), 2 M-segments (Medium), and 4 S-segments (Small) [48]. Based on the gene mutations of the Cσ protein, responsible for the adhesion of the virus to the host, the five genotypic groups of ARVs are determined, varying their pathogenic effect from only tendosynovitis or enteric disease, to immunosuppression or malabsorption of food [34,46]. ARVs are often highly prevalent in commercial turkeys in a subclinical manner, and in some cases, mild symptoms such as lameness begin to be observed. It is usually seen in co-infection with other enteric pathogens, especially bacteria [7,46].

**Figure 3:**
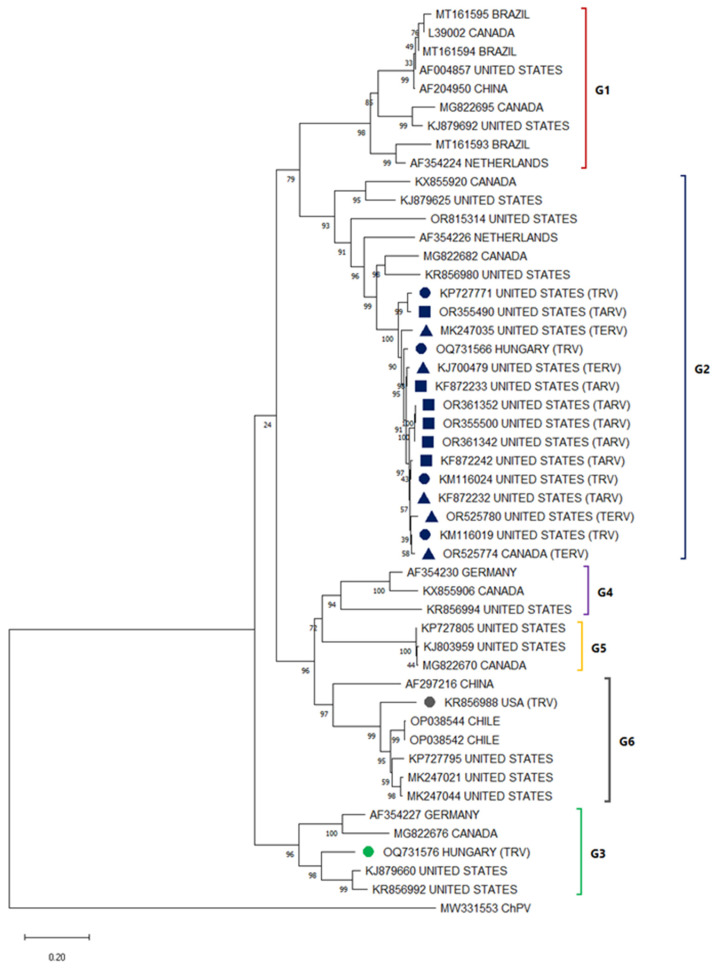
Phylogenetic relationships between ARV nucleotide sequences (some sequences from every location were selected randomly), including sequences isolated in infected turkeys (G1 in red, G2 in blue, G3 in green, G4 in purple, G5 in yellow and G6 in gray) based on Cσ gene sequences previously deposited in the GenBANK distributed by the countries in which they were reported. The sequences were aligned using the CLUSTAL OMEGA method in ClustalX2 2.1. The phylogenetic tree was constructed using the MEGA 11 software package using the maximum likelihood statistic method and the Tamura-Nei substitution model. The numbers along the branches refer to bootstrap values for 1000 replicates. The scale bar represents the number of substitutions per site. TRV sequences reported in cases of arthritis or tendosivitis (TARV) were marked with a square. TRV sequences reported in cases of enteritis or liver disease (TERV) were marked with a triangle. TRV sequences reported in cases sharing both symptomologies were marked with a circle. A sequence of ChPV (MW331553) was used as an outgroup.

Despite the high diversity of existing ARV genotypes, the majority of TRVs correspond to type 2, associated with both enteric and arthritis symptoms [49]. These ARVs remain clustered together and closer to each other than to isolated sequences from other infected birds (Figure 3). Although there is an ARV vaccine corresponding to this genotype, it does not appear to be generating the immunity necessary for disease prevention. A recent study in turkeys observed susceptibility to TRVs in young and long-lived turkeys, despite previous vaccination, including by maternally derived antibodies [46]. With the aim of developing a specific vaccine for TRVs, a study in 2015 proposed a genotypic classification based on the genes of the M1, M2, and M3 segments, identifying 5, 7, and 3 genotypes, respectively [18]. This differentiation allowed separating TARVs and TERVs into distinct groups, establishing an independent classification of chicken reoviruses and highlighting the need to study separately the reoviruses of these species [18].

### 3.4. Avian Parvovirus

Avian parvoviruses are viruses that affect various species of birds, causing diseases that can compromise development, intestinal health, and the immune system. They are transmitted mainly by contact with contaminated feces and can persist in the environment for long periods [50]. Turkey parvovirus (TuPV) belongs to the family *Parvoviridae* and the genus *Aveparvovirus*, having been identified for the first time in 1983 in turkeys showing dwarfism, enteritis, and high mortality [51]. This virus has a single-stranded DNA genome of approximately 5.2 kb, which is divided into three ORFs: the NS, which codes for the non-structural protein NS1; the NP, which codes for the non-structural protein NP1; the VP, which codes for the overlapping capsid proteins VP1 and VP2, which are highly variable regions that confer specific pathogenic effects and differentiate it from other APV species [52]. Studies in the United States show a 78% prevalence of TuPV in wild and commercial turkey populations [12]. In addition to the common symptoms of enteritis and Turkey Dwarfism Syndrome associated with TuPV, this virus has been found in cases of cerebral hypoplasia and hydrocephalus in turkeys. The occurrence of parvovirus in turkeys has initially been linked to transmission from ducks, where the presence of TuPV is associated with dwarfism and broken beak syndrome (SBDS), as well as Derzsy’s disease, characterized by locomotor ataxia, anorexia, and growth retardation [53].

**Figure 4:**
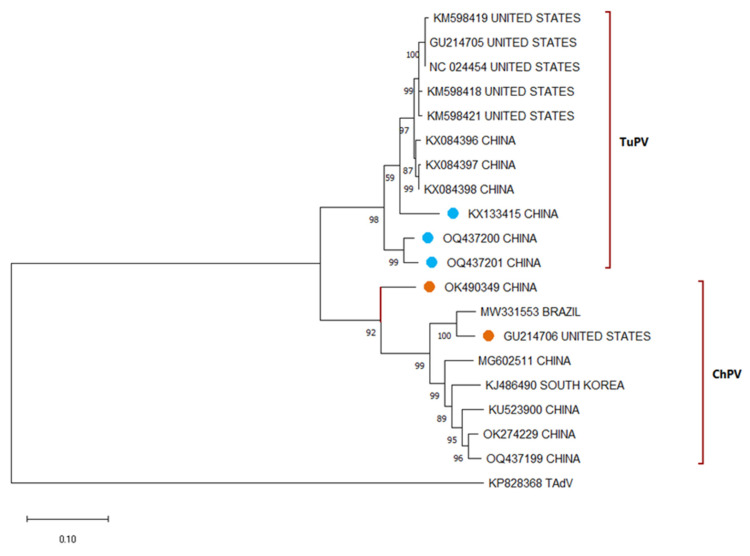
Phylogenetic relationships between APV nucleotide sequences (some sequences from every location were selected randomly), including sequences isolated in infected turkeys (TuPV in light blue and ChPV) based on VP1 sequences previously deposited in the GenBANK distributed by the countries in which they were reported. The sequences were aligned using the CLUSTAL OMEGA method in ClustalX2 2.1. The phylogenetic tree was constructed using the MEGA 11 software package using the maximum likelihood statistic method and the Tamura-Nei substitution model. The numbers along the branches refer to bootstrap values for 1000 replicates. The scale bar represents the number of substitutions per site. The ChPV sequences grouped in the TuPV sequence clade are marked with a light blue circle. TuPV sequences clustered in the ChPV sequence clade are marked with an orange circle. A sequence of TAdV (KP828368) was used as an outgroup.

The phylogenetic discrimination of TuPV and ChPV is complicated because they have a high genetic similarity, and it is hypothesized that they are evolutionarily very close, being able to be two genotypes of the same virus [16,54,55]. Using the VP region, it is possible to discriminate between these two viruses; however, sometimes sequences of one with fragments highly similar to the other have been observed, which have been proposed as recombinant strains [17], which are still unknown if they have a pathogenic effect for both bird species (Figure 4). Unlike ChPV, which has been classified into subgroups based on its pathogenic effect, TuPV strains have no specific classification. In Turkey, a new and emerging parvovirus was identified (*Chaphamaparvovirus galliform*). Although its effect on poultry houses has not yet been evaluated, it is a wake-up call for periodic study of these viruses [56].

### 3.5. Avian Adenovirus

Avian adenoviruses are viruses that affect different species of birds, where in the case of turkeys, they can cause diseases that compromise the immune, hepatic, and hematological systems [57]. They are transmitted mainly by feces and contaminated surfaces, in addition to vertical transmission, which facilitates their dissemination in poultry houses [57,58]. Turkey Adenovirus (TAdV) belongs to the family *Adenoviridae* and the genus *Siadenovirus*. It is a syngeneic virus with a double-stranded DNA genome of between 26 and 45 kb, depending on the viral strain; therefore, the number of ORFs in this virus is highly variable and is divided into early regions (E1-E4) and late regions (L1-L5) [59]. The late L1 region contains the hexon gene, which is the structural protein of the viral capsid, the most variable region of the genome, and the one used to differentiate the types of TAdV [60]. At least five types of TAdV have been proposed, which are similar to the adenoviruses that infect chickens (FAdV A-E). Among the TAdV types, the most relevant are TAdV-1 and TAdV-3, sometimes called hemorrhagic enteritis virus (HEV) [58,61]; where the former is associated with turkey spleen syndrome that produces splenomegaly and immunosuppression; while the latter is a causative agent of hemorrhagic enteritis in turkeys, also called marbled spleen disease, causing bloody diarrhea, anemia, splenomegaly and high mortality in young turkeys [34,62,63].

**Figure 5:**
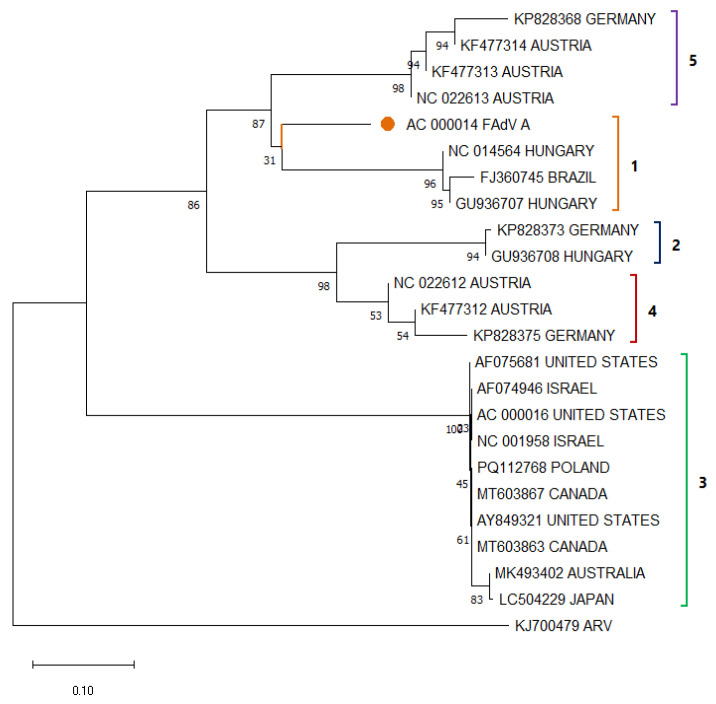
Phylogenetic relationships between FAdV nucleotide sequences (some sequences from every location were selected randomly), including sequences isolated in infected turkeys (TAdV-1 in orange, TAdV-2 in blue, TAdV-3 in green, TAdV-4 in red, and TAdV-5 in purple) based on Hexon sequences previously deposited in the GenBANK distributed by the countries in which they were reported. The sequences were aligned using the CLUSTAL OMEGA method in ClustalX2 2.1. The phylogenetic tree was constructed using the MEGA 11 software package using the maximum likelihood statistic method and the Tamura-Nei substitution model. The numbers along the branches refer to bootstrap values for 1000 replicates. The scale bar represents the number of substitutions per site. One FAdV-A sequence was included and circled in orange. A sequence of ARV (KJ700479) was used as an outgroup.

Among the different types of TAdV and FAdV, there is usually high genetic variability, being divided into phylogenetic clades (Figure 5); however, the reported strains of the same group usually maintain high percentages of similarity, especially TAdV-3 strains [61,64]. Despite the high diversity of HEV sequences reported around the world, they have a low genetic variability among them, remaining very close in nucleotide-based phylogenetic analyses; however, transcriptomes seem to have significant variations, which may increase the pathogenic effect [61]. Recently, a strain of FAdV-8b was found in turkeys with enteric disease in Egypt, which caused inclusion body hepatitis characteristic of this virus, exacerbating the need for epidemiological surveillance of this group of viruses [65].

### 3.6. Turkey Coronavirus

Avian coronaviruses are viruses that affect various species of birds, causing respiratory, digestive, and reproductive diseases. They are mainly transmitted by direct contact with infected secretions and can have a significant impact on poultry farming [66]. Their distribution is global, and some variants can cause severe outbreaks on poultry farms. Turkey coronavirus (TCoV) is an enveloped virus, belonging to the family *Coronaviridae* and the subfamily Orthocoronavirinae, with a single-stranded RNA genome of approximately 27 kb [67,68]. Its genome is divided into 11 ORFs encoding 15 non-structural proteins encoded in Open Reading Frame (ORF) 1a and 1b at the 5′ end, together with structural proteins of the envelope (E), membrane (M), nucleocapsid (N) and spike (S), plus other low molecular weight proteins that vary by subgenus. The coding region for the S protein comprises the most variable part of the coronavirus genome and is used for genotype differentiation [67]. Although first described in 1970 [69], TCoV was first isolated in 2008 from turkeys affected by PEC [70]. TCoV infections have been associated with enteritis, defects in eggshell formation, and respiratory disease, resulting in a high probability of sudden death from PEMS in turkeys up to 5 weeks of age [71]. Its pathology is characterized by ruffled feathers, reduced feed intake, and wet greenish feces with severe mucus, leading to PEMS in up to 9% of cases [72]. Both enteric and non-enteric pathology strains have been shown to possess tropism for intestinal tissues, providing them with the ability to replicate rapidly in these tissues [73].

**Figure 6:**
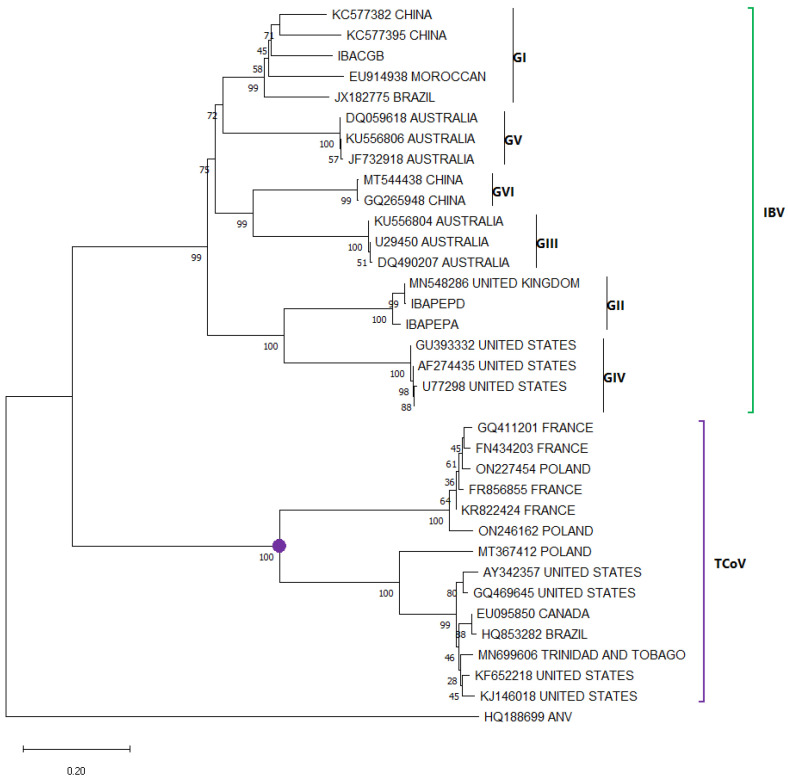
Phylogenetic relationships between AvCoV nucleotide sequences (some sequences from every location were randomly selected), including sequences isolated in infected turkeys (IBV in green and TCoV in purple), based on S1 sequences previously deposited in the GenBANK distributed by the countries in which they were reported. The sequences were aligned using the CLUSTAL OMEGA method in ClustalX2 2.1. The phylogenetic tree was constructed using the MEGA 11 software package using the maximum likelihood statistic method and the Tamura-Nei substitution model. The numbers along the branches refer to bootstrap values for 1000 replicates. The scale bar represents the number of substitutions per site. The TCoV clade is marked with a purple circle. A sequence of ANV (HQ188699) was used as an outgroup.

Unlike AvCoV, which infects chickens (IBV) and has been classified into six genotypes (Figure 6), TCoV does not have a defined phylogenetic classification. Despite its phylogenetic closeness, TCoV does not appear to share genotypes with IBV. In the case of the latter, even within the GI genotype, at least 27 IBV lineages have been identified, which cause diverse pathologies from subclinical symptoms to high mortality rates [74]. Possible cases of recombination between IBV and TCoV strains have been evaluated, although their pathological effects have not been fully evaluated, this could indicate a behavior or relationship with the genetic distribution of IBV and in the future could be used as a reference for the grouping of TCoV strains into genotypes or lineages using new and accurate models for investigation [20,21,75]. A 2015 study in the United States proposed four genotypic groups based on locality and nucleotide similarity at S1; however, it does not take into consideration the unique pathogenic effect of each group, is not feasible when analyzing amino acids and does not take into account strains reported in Poland, Brazil and Trinidad and Tobago; which do not follow the same behavior [76]. Extensive phylogenetic studies are needed to definitively define the possible groups of this virus.

**Table 1:** Reported prevalence (%) of turkey enteric viruses, including Turkey Astrovirus (TAstV), Turkey Rotavirus (TRotV), Turkey Reovirus (TRV), Turkey Parvovirus (TuPV), Turkey Adenovirus (TAdV), and Turkey Coronavirus (TCoV), based on studies conducted in different countries and continents. Detection methods are indicated by symbols: ● Molecular techniques (e.g., RT-PCR, qPCR), □ Electron microscopy, ▲ ELISA, ◊ and Virus isolation. Asterisks (*) denote statistically significant differences when compared to other regional data. UTA = Unclassified Turkey Astrovirus; UTAd = Unclassified Turkey Adenovirus; UV = Unstudied Virus (no reports available).

Locality		Enteric Viruses Prevalence											
		TastV		TRotV		TRV		TuPV		TAdV		TCoV	
**South America**	**Brazil**	30% (ANV) ^●^ 10% (TAstV-1) ^●^ 44.7% (TAstV-2) ^●^	[11,77]	52.60% ^●^	[11]	7.9% ^●^	[11]	N/A	N/A	5.3% (TAdV-1) ^●^ 0% (HEV) ^●^	[11]	55.30% ^●^	[11]
**North America**	**United States**	74.4%(UTA) □ 55.8% (TAstV-2) ^●^ 9.3% (TAstV-1) ^●^	[78]	51.2% ^●^	[79]	53.5% □	[80]	2.3% ^●^	[12]	63.15%(HEV) ^●^	[81]	10.03% ^●^	[80]
	**Canada**	UV	UV	UV	UV	UV	UV	UV	UV	43.75%(HEV) ^●^	[63]	UV	UV
**Europe**	**Poland**	44.90% (UTA) ^●^ 35.3% (TAstV-1) ^●^ * 94.1% (TAstV-2) ^●^	[82,83]	18.8% ^●^	[84]	77.50% ◊	[84]	27.5% ^●^	[83]	8.98% (UTAd) ^●^	[85]	9.7% ^●^	[86]
	**Germany**	UV	UV	* 88.15% ^●^	[87]	UV	UV	UV	UV	30.43%(UTAd) ◊	[88]	UV	UV
	**Czech Republic**	UV	UV	UV	UV	UV	UV	UV	UV	65.07% (HEV) ▲	[89]	UV	UV
**Asia**	**Turkiye**	43.4% (TAstV-2) ^●^	[90]	UV	UV	UV	UV	70% ^●^	[17]	UV	UV	UV	UV
	**Iran**	UV	UV	UV	UV	UV	UV	UV	UV	UV	UV	44.4% ^●^	[91]
	**Hungary**	4.08% (ANV) ^●^ 83.67% (TAstV-1) ^●^ 26.53% (TAstV-2) ^●^	[14]	28.57% ^●^	[14]	14.28% ^●^	[14]	73.46% ^●^	[14]	0%(HEV) ^●^	[14]	14.28% ^●^	[14]
	**China**	UV	UV	UV	UV	UV	UV	85.71% ^●^	[53]	UV	UV	UV	UV
**Oceania**	**Australia**	UV	UV	UV	UV	UV	UV	UV	UV	80% ▲	[92]	UV	UV
**Africa**	**Nigeria**	0% ^●^	[93]	0.00% ^●^	[93]	0.00% ^●^	[93]	UV	UV	0.00% ^●^	[93]	0% ^●^	[93]

### 3.7. Last Reported Prevalence Within the Last 15 Years

Although the presence of these viruses is assumed in several countries, such as Poland, Germany, the United States, Brazil, the Netherlands, Italy, among others, few of them have updated data on the prevalence of these viruses in turkey farms, as well as the presence of specific genotypes. In North America and Europe, at least one AAstV was the most prevalent virus in these continents (Table 1). In the second case, Poland had the highest positivity ratio for any of the viruses tested, with 94.1%, showing significant differences with the other viruses in this region, with the exception of TRV from the same country and TRotV in Germany. Due to its easy transmission (vertical and horizontal), the spread of these poultry viruses is fast and difficult to control, which is why it is usually the highest in poultry farms. Among the frequencies of TRotV obtained, the highest prevalence was identified in Germany, with 88.15% in 2011, showing significant differences among the rates of this virus reported in all other continents. As for reports of TuPV throughout the world, Asia has the highest rates, where a study in 2023 in China reported a high distribution in poultry houses caused by new strains (with 85.71% positive samples). Several other reports have been made in the years prior to the papers reported, where, in most cases, the prevalences have remained the same or increased, indicating that the problem has not been resolved over the years [94,95,96].

### 3.8. Coinfections Involving Turkey Enteric Viruses

Co-infections between enteric cirrhosis in turkeys are recognized as an important factor in the pathogenesis and epidemiology of turkey cirrhosis in relation to PEC [2,97]. The prevalence of coinfections can vary considerably depending on the region in which they occur and all of the unique factors involved. In the United States, TuPV was detected in 71.6% of samples, with frequent coinfections involving small round viruses observed by electron microscopy [15]. In Brazil, over 93% of flocks tested positive for at least one virus, with the most common coinfections involving ARV, CAstV, and ChPV [11]. A 2024 study confirmed these patterns, finding that only 6% of samples were free of viral agents [98]. In China, TuPV was found in 83.3% of farms, with the most frequent coinfection being TuPV–ChPV [99]. These findings emphasize the importance of considering coinfections in PEC diagnosis and control strategies.

### 3.9. Other Viruses

Among the new viral agents associated with PEC, avian picornaviruses have received attention. A study published in 2014 identified new picornaviruses in turkeys with clinical signs of enteritis [100]. These viruses belong to the family Picornaviridae and have been classified into genera such as Sapelovirus and Gallivirus; in 2015, a prevalence of 57.5 was reported in cases of turkeys with LTS [13]. Another group of emerging viruses is the avian caliciviruses. Recent investigations have detected the presence of caliciviruses in turkey samples with enteritis. These viruses belong to the Caliciviridae family and have been associated with enteric diseases in various animal species [80,100]. In addition, new viruses of the Picornaviridae family have been identified in turkeys with enteritis. A study published in 2016 reported the detection of an avian picornavirus in turkeys with clinical signs of enteritis [100,101]. This finding suggests that these viruses may play a role in the pathogenesis of PEC, although further studies are required to confirm their involvement.

### 3.10. Cross-Species Infections of Enteric Viruses

Although these enteric viruses are primarily found in turkeys, recent studies have documented their presence in other avian hosts. TAstV-1 has been identified in asymptomatic Brazilian chickens for the first time, suggesting possible cross-species transmission (six out of 60 pooled samples) [94]. Although TCoV is largely non-pathogenic in chickens, it can replicate in their intestinal and respiratory tissues following experimental inoculation, indicating a potential carrier role without visible disease [102]. TuPV/ChPV have recently been detected in both turkeys and chickens, including mixed infections on Turkish farms, with 72.3% positivity in broilers and 70% in turkeys [17]. Phylogenetic analyses reveal shared lineages and evidence of recombination across species [54]. Finally, TAstV and related astroviruses have been found in multiple avian species, including ducks, guinea fowl, and wild birds, highlighting their endemic circulation beyond turkeys [103].

### 3.11. Clinical Features of Enteric Viral Infections in Turkeys

Enteric viral infections in turkeys present with a variety of clinical signs, which depend on factors such as age, co-infections, and environmental conditions [104]. TAstV infection in young poults typically causes diarrhea, stunted growth, poor weight gain, and litter dehydration [80]. Experimental TAstV-2 infections have been shown to cause severe enteritis, crypt hyperplasia, and reduced body weight in one-week-old poults [105]. TRotV, particularly group A, has been associated with watery diarrhea and lethargy, with affected flocks demonstrating reduced feed efficiency and uneven growth [106]. Mixed infections with other viruses or bacteria exacerbate the clinical signs [106]. TRV has been associated with enteric symptoms, immunosuppression, and malabsorption [102]. Field reports have noted increased mortality, poor flock uniformity, enterocyte degeneration, and lymphoid depletion [107,108]. TuPV is often associated with RSS in poults under four weeks old and causes diarrhea, poor feathering, and growth disparity [97]. TCoV causes transmissible enteritis in both young and adult turkeys. It is characterized by yellow to green watery diarrhea, dehydration, rapid weight loss, and, occasionally, increased mortality [109]. Histopathological changes include villus atrophy and crypt hyperplasia, mainly in the jejunum and ileum [109]. TAdVs are usually subclinical, but have been implicated in enteritis outbreaks [80]. Some fowl adenovirus serotypes have been linked to inclusion body hepatitis and gizzard erosion, particularly in cases of co-infection [110]. Together, these viruses contribute to the clinical presentation of PEC, which is characterized by diarrhea, uneven growth, an elevated feed conversion ratio, and, in severe cases, high morbidity and mortality [97]. Clinical signs are often non-specific and overlapping, which complicates field diagnosis and highlights the importance of molecular confirmation [8].

## 4. Diagnostic Methods

### 4.1. PCR/qPCR and NGS

Polymerase Chain Reaction (PCR) and its variants, such as quantitative PCR (qPCR), are widely used due to their high sensitivity and specificity in detecting viral genetic material. These methodologies have proven to be effective in the identification of viruses, such as rotaviruses, astroviruses, and coronaviruses, in turkey fecal samples, providing valuable information for epidemiological studies and control programs. In the case of RNA viruses, the Reverse Transcriptase PCR (RT-PCR) technique becomes an essential tool, as it allows the conversion of viral RNA into complementary DNA prior to amplification, facilitating the detection of pathogens such as avian coronaviruses [78,111,112]. Among the most prominent published assays is a multiplex RT-PCR assay from 2007 that includes the simultaneous detection of TAstVs (ANV and TAstV-1 and 2) and AvRVA, although with detection limits that do not reach one copy [112]. Among the most recent studies focused on the detection of turkey enteric viruses, one based on qPCR with TaqMan hydrolysis probes from 2020 was designed for the detection of TRV, and another for HEV in 2013 and continuously used [81,113]. Recently, in 2022, a study was published that described a tri-plex qPCR assay with TaqMan hydrolysis probes for the detection of TAstVs; however, the validation of this is not complete, and assays are lacking to prove its effectiveness [78]. Finally, PCR assays capable of detecting viruses of different species have been described for coronaviruses and astroviruses, whose application has allowed the detection of new species of these viruses [114,115]. Next generation sequencing (NGS) is sometimes used as a form of detection for virus due to the high information it provides, complete sequences; however, its high cost is not very productive for epidemiological studies with a high number of samples and its application is mostly focused on identifying divergent from previously re-carried samples or to identify new pathogens associated with a specific disease [116,117].

### 4.2. ELISA and Serological Techniques

An ELISA is a widely used technique that allows the detection of specific antibodies or viral antigens in sera or fecal samples, which facilitates the identification of previous or ongoing infections and provides key information for epidemiological surveillance [118,119]. Its sensitivity and capacity to process large volumes of samples make it an efficient method for population studies and health surveillance [118]. On the other hand, IFA (Immunofluorescence Assay) is used in the detection of specific antibodies against enteric viruses in turkey sera, being a valuable technique in serological epidemiological studies and in the confirmation of active infections [120]. This methodology, based on the binding of fluorescent antibodies to viral antigens, allows the visualization of the antigen-antibody interaction by fluorescence microscopy, providing a complementary tool for the immunological characterization of infections in poultry farms [121]. Serological methods have been previously documented for the six viruses mentioned, although their initial application was primarily focused on chickens; this has since been extended to turkeys; however, most of these were described more than 15 years ago causing that their effectiveness over time is not clear and they have been replaced by molecular methods in the context of diagnosis [80,118,119,120,121,122,123].

### 4.3. Electron Microscopy

Transmission Electron Microscopy (TEM) allows direct visualization of viral particles in fecal samples, which is particularly useful for the detection of viruses such as rotaviruses and astroviruses [25,124]. Although its sensitivity is lower compared to molecular techniques, TEM provides valuable morphological information that facilitates the identification and classification of viruses present in samples [102]. On the other hand, viral isolation in cell culture remains an important technique for pathogen characterization, allowing virus replication in avian cell lines and enabling neutralization tests to assess pathogenicity [50,80]. Despite these limitations, both techniques remain relevant in virological studies requiring structural confirmation or analysis of new viral variants in the poultry industry. Even taking into account its usefulness, electron microscopy alone is not applicable as a diagnostic method, and, even in those cases where it is applied, the preference is to confirm with molecular methods.

## 5. Control and Prevention Strategies

Strict biosecurity measures, such as access control, facility disinfection, and proper waste management, are critical to preventing the introduction and spread of infectious agents while optimizing the environment, including ventilation and stocking density, while reducing stress and strengthening the immune system of the birds. Targeted vaccination programs against specific pathogens, such as adenoviruses and reoviruses, have been shown to reduce the incidence and severity of infections, provided they are based on local epidemiology [125,126]. Diet also plays a key role, as a balanced diet supplemented with probiotics and prebiotics supports the gut microbiota and strengthens resistance to infection, and control of mycotoxins in feed is crucial to avoid immunosuppressive effects [127,128,129]. Continuous monitoring and early diagnosis using accurate techniques allow rapid detection of pathogens, which facilitates the implementation of timely corrective measures and minimizes economic losses.

## 6. Conclusions

PEC is a multifactorial syndrome in turkeys caused by a diverse range of enteric viruses, including astroviruses, rotaviruses, reoviruses, parvoviruses, adenoviruses, and coronaviruses, which affect digestive health, nutrient absorption, and overall growth. These viruses are transmitted through fecal contamination and, in some cases, vertically, persisting in the environment and facilitating widespread infection. Co-infections with multiple viruses are common, further complicating disease presentation and severity. Diagnostic approaches range from molecular methods like PCR/qPCR and RT-PCR/RT-qPCR, serological assays, such as ELISA and immunofluorescence, to traditional techniques like electron microscopy and viral isolation. Prevention and control strategies emphasize strict biosecurity measures, vaccination programs, optimized nutrition, and responsible antibiotic use to mitigate the impact of PEC. A comprehensive, multidisciplinary approach combining early detection and proper management is essential for controlling PEC and reducing its economic impact on the poultry industry. This approach could be used in the future for the manufacture of specific vaccines for those viruses that have not yet developed immunity, thus allowing adequate immunization essential for disease prevention.

## Data Availability

Not applicable.

## Abbreviations

The following abbreviations are used in this manuscript:

PEC	Poult Enteric Complex
PEMS	Poult Enteritis and Mortality Syndrome
TMS	Turkey Mortality Syndrome
RSS	Runting-Stunting Syndrome
LTS	Light Turkey Syndrome
PES	Poult Enteritis Syndrome
AAstV	Avian Astrovirus
TAstV	Turkey Astrovirus
ANV	Avian Nephritis Virus
AvRV	Avian Rotavirus
TRotV	Turkey Rotavirus
AvRVA	Avian Rotavirus Group A
ARV	Avian Reovirus
TRV	Turkey Reovirus
TARV	Turkey Arthritis Reovirus
TERV	Turkey Enteric Reovirus
APV	Avian Parvovirus
TuPV	Turkey Parvovirus
ChPV	Chicken Parvovirus
TAdV	Turkey Adenovirus
FAdV	Fowl Adenovirus
HEV	Hemorrhagic Enteritis Virus
TCoV	Turkey Coronavirus
AvCoV	Avian Coronavirus
IBV	Infectious Bronchitis Virus
AvPiV	Avian Picornavirus
ACV	Avian Calicivirus
PCR	Polymerase Chain Reaction
qPCR	Quantitative PCR
RT-PCR	Reverse Transcriptase PCR
NGS	Next-Generation Sequencing
ELISA	Enzyme-Linked Immunosorbent Assay
IFA	Immunofluorescence Assay
TEM	Transmission Electron Microscopy
ORF	Open Reading Frame
RdRp	RNA-dependent RNA Polymerase
VP	Viral Protein
NSP	Non-Structural Protein
